# Differential effects of PCSK9 inhibitors and statins on plasma ceramides in coronary artery disease

**DOI:** 10.3389/fphar.2025.1726925

**Published:** 2025-12-18

**Authors:** Liang Zhang, Yaodong Ding, Yong Zeng

**Affiliations:** Department of Cardiology, Beijing Anzhen Hospital, Beijing Institute of Heart, Lung and Blood Vessel Disease, Capital Medical University, Beijing, China

**Keywords:** PCSK9 inhibitors, statin, ceramide, coronary artery disease, lipid

## Abstract

**Background:**

While elevated plasma ceramides are independently associated with increased cardiovascular risk in patients with coronary artery disease (CAD), the impact of current lipid-modifying drugs on ceramides, remains under-researched. This study examines the effect of PCSK9 inhibitors on plasma ceramides in patients with CAD who had received statin therapy.

**Methods:**

Comparing the effect of PCSK9i vs. statin on ceramide levels. The primary outcome was percent change in ceramide levels. Subgroup analyses were done to explore potential treatment effect differences.

**Results:**

Among 292 patients (44% statin group, 56% PCSK9i group), baseline characteristics were broadly similar. PCSK9i use significantly reduced ceramide levels compared to statin therapy: Cer 16:0 decreased by −22.93% (95% CI, −29.73% to −16.14%), Cer 18:0 by −24.54% (95% CI, −33.07 to −16.01), and Cer 24:0 by −34.82% (95% CI, −48.41 to −21.23). PCSK9i also significantly lowered LDL-C by 29.63%, triglycerides by 16.69%, and total cholesterol by 10.25%, while modestly increasing HDL-C. Sensitivity and subgroup analyses confirmed the consistent ceramide-lowering effect of PCSK9i across various patient demographics and baseline characteristics.

**Conclusion:**

PCSK9i is associated with significant reduction in distinct ceramide concentrations, compared statin therapy only.

## Highlights

- We demonstrate that PCSK9i therapy significantly reduces distinct ceramide species (Cer 16:0, Cer 18:0, Cer 24:1 and Cer 24:0), which are independently associated with increased cardiovascular risk in patients with coronary artery disease.
- Our findings highlight the potential clinical implications of PCSK9 inhibitors in reducing cardiovascular risk beyond LDL-C lowering.
- Our study addresses a significant gap in the current literature by investigating the differential impact of PCSK9 inhibitors and statins on plasma ceramide levels in patients with coronary artery disease.

## Introduction

An increasing body of research has identified plasma ceramides as important prognostic markers in patients with coronary artery disease (CAD) (Choi et al., 2021). Ceramides are bioactive molecules involved in various cellular processes, and trials in rodents have demonstrated that the disease condition can be improved by modulating the ceramide profile (Chaurasia et al., 2019; Summers et al., 2019; Bharath et al., 2015). Recent study also demonstrated that the effect of Cer16:0 - CYSLTR2/P2RY6 - GPCRs (G-protein-coupled receptors) signaling on the progression of atherosclerosis in both humans and mice (Zhang et al., 2025). Targeting ceramide lowering therapy may be a key breakthrough in future anti-atherosclerotic treatment.

Lipid-lowering therapy is essential in the treatment of coronary heart disease (Mach et al., 2025). PCSK9, a protein generated by the liver, primarily regulates the levels of low-density lipoprotein cholesterol in the blood by influencing the degradation of LDL-C receptors and acts as a vital part of this process (Emma et al., 2020). A *post hoc* analysis of the ODYSSEY OUTCOMES trial using the baseline apolipoprotein profile for high-risk assessment can indicate the potential benefits that patients will receive from the alirocumab (Reijnders et al., 2025). This suggests that PCSK9 inhibitors (PCSK9i) might be effective in reducing residual risk by diminishing other lipid molecules, in addition to its impact on LDL-C (O'Donoghue et al., 2019; Klug et al., 2024). Interestingly, lipid-lowering medication such as statins and PCSK9 inhibitors also reduce ceramide levels (Ng et al., 2014; Tarasov et al., 2014; Ye et al., 2020). Additionally, rosuvastatin dose-dependently decreases plasma ceramide—independent of low-density lipoprotein (LDL) reduction—in men with metabolic syndrome (Ng et al., 2014). A previous study showed that PCSK9 inhibitor treatment in 24 hypercholesterolemic patients led to a significant reduction in specific ceramides (Cer 16:0, Cer 18:0, Cer 24:1, and Cer 24:0), and the reduction in ceramides did not correlate with the reduction in LDL-C^13^. However, there are no direct comparisons available to evaluate the efficacy of the two lipid-lowering medication (Statin vs. PCSK9i), and the previous studies both suffered from small sample sizes.

We performed a study on retrospective clinical data to compare the effects of Statins and PCSK9 inhibitors on reducing the plasma ceramide concentrations in CAD patients. These effects may offer valuable therapeutic opportunities in CAD patients exposure to high ceramide level.

## Methods

### Study design and population

Patients diagnosed with CAD who underwent at least two plasma ceramide examinations at Beijing Anzhen Hospital, Capital Medical University, were screened between March 2023 and May 2025 (Patients with multivessel coronary artery disease undergo staged treatment of the affected vessels, with interventions spaced approximately 1 month apart). Exclusion criteria included: 1. patients previously treated with PCSK9i before enrollment; 2. patients with a time interval between serial ceramide measurements of less than 3 weeks or more than 6 weeks; 3. patients with incomplete clinical data regarding lipid-lowering treatment protocols. Study participants were categorized based on the initiation of PCSK9i therapy after the first ceramide examination (Treatment group: PCSK9i combined with statin, Placebo group: statin only or combined with ezetimibe). The primary efficacy endpoint was the percentage change in plasma ceramide from baseline to repeated measurement in the PCSK9i group compared with the placebo group. Baseline was defined as the last measurement prior to the first dose of PCSK9i. The study protocol was in accordance with the Declaration of Helsinki and was approved by the Medical Ethics Committee of Beijing Anzhen Hospital (IRB number: KS2023081), with informed consent obtained from all participants.

### Data collection

Upon initial discharge, these patients are prescribed two doses of PCSK9 inhibitors. We gathered patients over 4–5 weeks to ensure they received a consistent PCSK9i dosage (Evolocumab 140 mg/Alirocumab 75 mg i.h. Biw). The demographic variables comprised age and sex. Clinical and laboratory data encompassed 1. medical history pertaining to diabetes mellitus, hypertension, and ischemic stroke; 2. baseline and serial measurements of serum lipid levels, including total cholesterol (TC), low-density lipoprotein cholesterol (LDL-C), high-density lipoprotein cholesterol (HDL-C), triglycerides (TG), as well as plasma ceramide levels (Cer 16:0, Cer 18:0, Cer 24:1, and Cer 24:0); and 3. lipid-lowering medications administered during hospitalization, specifically statins, ezetimibe, and PCSK9 inhibitors.

### Measurement of ceramide level

Patients fasted overnight for 8 h before their scheduled ICA. On the morning of the initial and follow-up ICA visits, 500 μL blood samples were taken. Plasma was separated within an hour and stored at −80 °C for later analysis. Plasma samples were thawed at 4 °C, mixed with internal standards, and treated with isopropanol for protein precipitation. After vortexing and centrifuging at 4000 rpm for 15 min at 4 °C, the supernatant was transferred to a 96-well plate for ceramide analysis (Cer 16:0, Cer 18:0, Cer 24:1, and Cer 24:0) using an LC-MS/MS system. The methods used in the laboratory and the quality control procedures have been described in detail in prior reports (Zhang et al., 2023).

### Statistical analysis

The prespecified primary efficacy estimand involved an assessment of the mean treatment effect of PCSK9 inhibitors in comparison to placebo within a population that adheres to statin therapy. Post hoc statistical power was calculated for each ceramide species based on the observed effect size (Cohen’s d) derived from the group differences and the actual sample size of the study. All analyses demonstrated excellent statistical power (>0.94).

Continuous variables were presented as mean ± standard deviation or median (IQR) based on normality assessed by Shapiro-Wilk tests. Baseline characteristics were compared between treatment groups (Statin vs. PCSK9i plus statin) using independent t-tests (normal distributions), Mann-Whitney U tests (non-normal distributions). Categorical variables were presented as frequencies (percentages) and compared between groups using the chi-square test or Fisher’s exact test. Ceramide levels were assessed at baseline (T0) and 1-month follow-up (T1). Change values (Δ) were calculated as T1 – T0, relative reduction (Δ %) were calculated as (T1 –T0)/T0 × 100%. Analysis of covariance (ANCOVA) model with age as a covariate was conducted to assess the differences in the time-averaged percent change in ceramide levels from baseline between the PCSK9i and statin groups. For the pairwise comparisons between these groups, the least-squares (LS) means, standard errors, and two-sided 95% confidence intervals were calculated and reported. Moreover, Propensity Score Matching (PSM,genetic, 1:1) and Generalized Linear Mixed Model (model < - nlme::lme (Δ_Ceramide % ∼ PCSK9i + Age + Sex + Hypertension + Diabetes + Stroke + LDL-C_T0 + HDL-C_T0 + TG_T0 + TC_T0, random = ∼1|id) was conducted as a sensitivity analysis to assess the association between PCSK9 inhibitor treatment and percentage change in Ceramide. Subgroup analysis was carried out to confirm whether the association of PCSK9i with change in ceramide was consistent across all pre-specified subgroups (Sex, Hypertension, Diabetes and LDL-C concentration at baseline). Statistical analyses were conducted using R Programming Language 4.3.2 (Vienna, Austria). Treatment comparisons for primary end points were performed at the full significance level of *P* < 0.05.

## Results

### Patient characteristics

**FIGURE 1:**
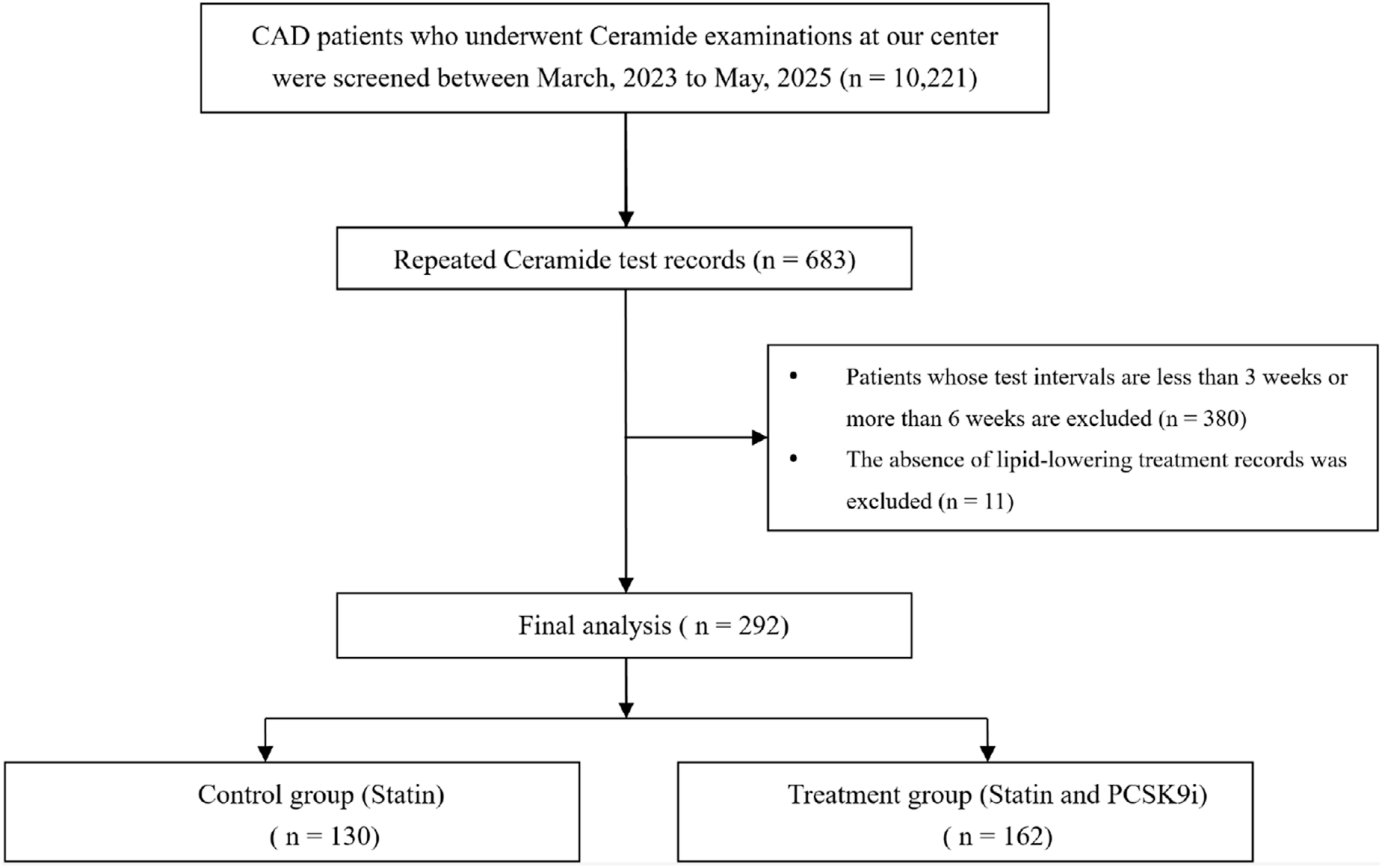
Flowchart of study process. Abbrevation: *CAD* coronary artery disease, *PCSK9i* proprotein convertase subtilisin/kexin type 9 inhibitors.

The baseline characteristics of the study population (N = 292), stratified by the use of PCSK9i at discharge, are presented in Table 1. Of these, 130 (44%) in the statin group and 162 (56%) in the PCSK9i group. The median age was 61 years, 58 participants (20%) were female. Among these patients, 181 participants (62.0%) had established hypertension, while diabetes (73.7%), stroke (6.1%) were prevalent. Baseline characteristics were broadly similar across groups. The most frequently used lipid-lowering medications were atorvastatin (44.6% vs. 59.3%), and rosuvastatin (50% vs. 38.9%). Ezetimibe use differed significantly between groups (31.5% vs. 0%, p < 0.001). The flowchart of patients enrolled are summarized in Figure 1.

**TABLE 1:** Demographic and clinical characteristics of the study cohort at baseline.

Characteristic	No. (%)	
	Statin n = 130	PCSK9i (+statin) n = 162
Age, median (IQR), years	61 (54, 67)	61 (53, 68)
Sex, n (%)		
Female	25 (19.2%)	33 (20.4%)
Male	105 (80.8%)	129 (79.6%)
Hypertension, n (%)	78 (60.0%)	103 (63.6%)
Diabetes, n (%)	56 (43.1%)	56 (34.6%)
Stroke, n (%)	5 (3.8%)	13 (8.0%)
Lipid-modifying therapy, n (%)		
Atorvastatin, 20 mg	58 (44.6%)	96 (59.3%)
Rosuvastatin, 10 mg	65 (50.0%)	63 (38.9%)
Other statin	7 (3.4%)	3 (1.8%)
Ezetimibe, 10 mg	41 (31.5%)	0 (0.0%)
Lipid measures at baseline, median (IQR), mmol/L		
LDL cholesterol	1.67 (1.31, 2.26)	2.42 (1.93, 2.97)
HDL cholesterol	1.06 (0.91, 1.23)	1.05 (0.92, 1.25)
Triglycerides	1.33 (0.92, 1.85)	1.67 (1.18, 2.40)
Total cholesterol	3.19 (2.75, 4.01)	4.06 (3.42, 4.76)
Ceramide measures at baseline, median (IQR), umol/L		
Cer 16:0	0.22 (0.17, 0.28)	0.25 (0.20, 0.32)
Cer 18:0	0.07 (0.05, 0.08)	0.08 (0.06, 0.12)
Cer 24:1	0.69 (0.54, 0.97)	0.85 (0.62, 1.07)
Cer 24:0	2.89 (2.18, 3.70)	3.50 (2.51, 4.75)
Cer 16:0/Cer 24:0	0.078 (0.060, 0.096)	0.073 (0.061, 0.089)
Cer 18:0/Cer 24:0	0.023 (0.016, 0.033)	0.022 (0.017, 0.032)
Cer 24:1/Cer 24:0	0.25 (0.18, 0.34)	0.24 (0.18, 0.32)

Abbreviations: HDL, high-density lipoprotein; LDL, high-density lipoprotein.

### Primary efficacy end point

**FIGURE 2:**
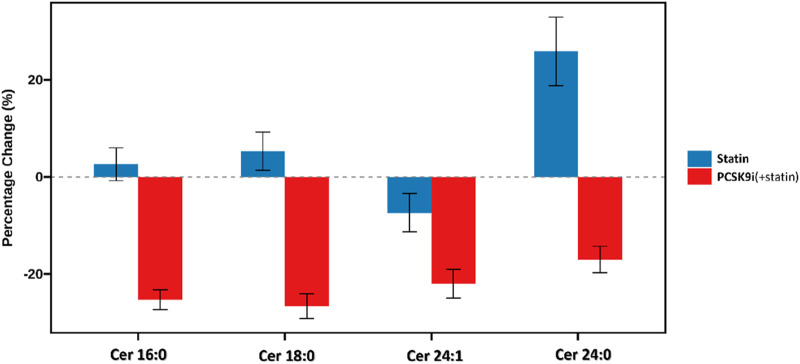
Percentage change in plasma ceramide concentrations after lipid-lowering therapy.

**FIGURE 3:**
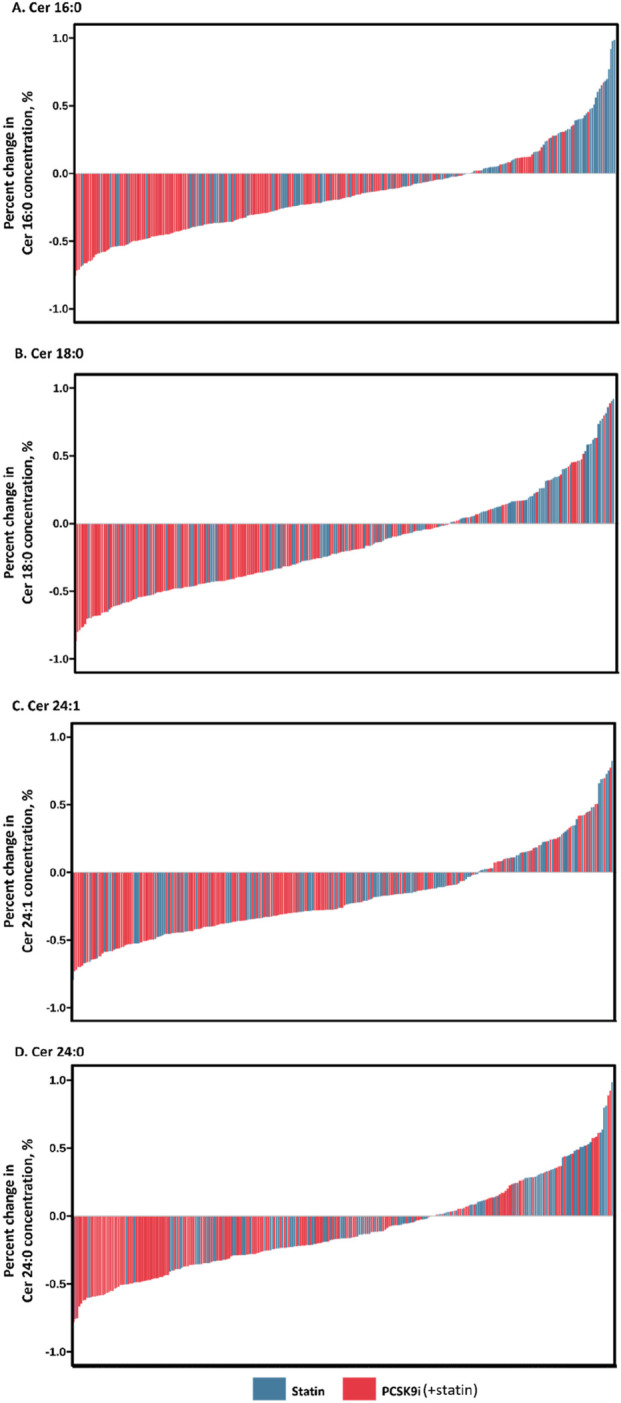
Percentage change in Ceramide concentration during month of treatment. Waterfall plots demonstrate individual absolute changes from baseline in Ceramide concentration (**(A)** Cer 16:0, **(B)** Cer 18:0, **(C)** Cer 24:1, **(D)** Cer 24:0) at month with statins and PCSK9 inhibitors.

**FIGURE 4:**
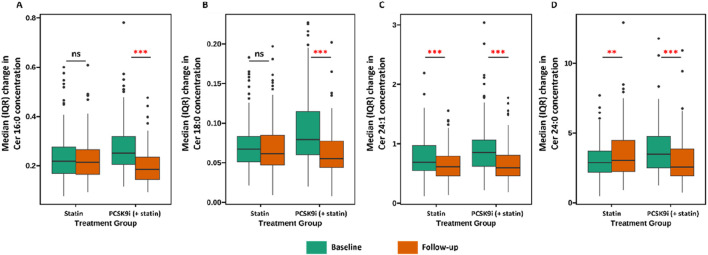
Association between treatment (Statin vs. PCSK9i) and changes in ceramide concentration (**(A)** Cer 16:0, **(B)** Cer 18:0, **(C)** Cer 24:1, **(D)** Cer 24:0). Data are presented as medians (lines), IQRs (boxes), 2.5th and 97.5th percentiles (whiskers), and outliers (solid dots). ** (p < 0.01), *** (p < 0.001), ns (p > 0.05).

The change in Ceramide from baseline with PCSK9i and statin for the primary efficacy end points are shown in Table 2 and Figure 2. The group that received PCSK9i showed a statin-adjusted time-averaged percent change in Cer 16:0 from baseline to 4 weeks of −22.93% (95% CI, −29.73% to −16.14%). For the Cer 18:0, least-squares mean differences between PCSK9i and placebo were −24.54 (95% CI, −33.07 to −16.01. For the Cer 24:0, the LS mean change from baseline compared to placebo was −34.82% (95% CI, −48.41 to −21.23). The treatment effects of PCSK9i and statins on Cer 24:1 lowering were not significantly different (p = 0.056), and the LS mean placebo-adjusted change from baseline with PCSK9i was −8.76% (95% CI, −17.7 to 0.19). The percentage change in Ceramide from baseline with different lipid lowering therapy in individual patients is shown as a waterfall plot in Figure 3. The absolute reduction from baseline in Ceramide with PCSK9i and statin treatment only are shown in Figure 4. Changes in other atherogenic lipids are shown in Table 2 with significant mean reductions relative to statin of 29.63% in LDL-C, 16.69% in triglycerides, and 10.25% in total cholesterol. PCSK9i and statins increased high-density lipoprotein cholesterol by 3.83%, and 2.12%, respectively.

**TABLE 2:** Percent changes in ceramides and other lipid parameters.

Variable	Statin, %change	PCSK9i (+statin), %change	Statin-adjusted change, LS mean % (95%CI)	P value
Ceramide measures at baseline, mean (SD), umol/L				
Cer 16:0	0.20 (0.10)	0.30 (0.10)	NA	NA
LS mean change^a^	−0.15	−23.09	−22.93 (−29.73 to −16.14)	<0.001
LS mean change^b^	−2.59	−25.51	−22.91 (−32.36 to −13.46)	<0.001
Cer 18:0	0.10 (0.03)	0.10 (0.05)	NA	NA
LS mean change^a^	1.19	−23.35	−24.54 (−33.07 to −16.01)	<0.001
LS mean change^b^	5.52	−22.89	−28.42 (−39.00 to −17.84)	<0.001
Cer 24:1	0.80 (0.35)	0.90 (0.45)	NA	NA
LS mean change^a^	−10.66	−19.41	−8.76 (−17.70 to 0.19)	0.056
LS mean change^b^	−5.79	−17.18	−11.40 (−22.08 to −0.72)	0.032
Cer 24:0	3.10 (1.29)	3.80 (1.71)	NA	NA
LS mean change^a^	21.37	−13.45	−34.82 (−48.41 to −21.23)	<0.001
LS mean change^b^	21.92	−14.82	−36.75 (−51.58 to −21.92)	<0.001
Lipid measures at baseline, mean (SD), mmol/L				
LDL cholesterol	1.80 (0.72)	2.50 (1.06)	NA	NA
LS mean change^a^	−4.22	−33.85	−29.63 (−40.27 to −18.98)	<0.001
HDL cholesterol	1.10 (0.29)	1.10 (0.26)	NA	NA
LS mean change^a^	3.83	2.12	−1.71 (−8.71 to 5.29)	0.632
Triglycerides	1.60 (1.24)	2.10 (1.53)	NA	NA
LS mean change^a^	6.9	−9.79	−16.69 (−25.43 to −7.96)	<0.001
Total cholesterol	3.40 (0.89)	4.20 (1.30)	NA	NA
LS mean change^a^	−8.78	−19.04	−10.25 (−19.99 to −0.52)	0.040

Abbreviations: HDL, high-density lipoprotein; LDL, high-density lipoprotein; LS, least-squares.

^a^ ANCOVA, model.

^b^ Generalized Linear Mixed Model.

### Sensitivity analyses

After PSM, 122 patients in the PCSK9i group and 122 patients in the statin group were selected (Supplementary Table 1; Supplementary Figure 1). The use of PCSK9i was associated with an absolute reduction from baseline in Ceramide compared with those with statin only (Table 3). Additionally, for the Generalized Linear Mixed Model, the effect of PCSK9i on ceramide reduction was consistent and independent of age, sex, hypertension, diabetes, and baseline values.

**TABLE 3:** Percent changes in ceramides and other lipid parameters after PSM.

Variable	Statin, %change	PCSK9i (+statin), %change	Statin-adjusted change, LS mean % (95%CI)	P value
Ceramide measures at baseline, mean (SD), umol/L				
Cer 16:0	0.20 (0.10)	0.30 (0.11)	NA	NA
LS mean change^a^	−1.75	−20.7	−18.95 (−26.07 to −11.83)	<0.001
Cer 18:0	0.10 (0.03)	0.10 (0.04)	NA	NA
LS mean change^a^	1.53	−21.65	−23.18 (−32.67 to −13.70)	<0.001
Cer 24:1	0.80 (0.35)	0.90 (0.48)	NA	NA
LS mean change^a^	−10.8	−19.26	−8.46 (−17.59 to 0.66)	0.070
Cer 24:0	3.10 (1.27)	3.90 (1.65)	NA	NA
LS mean change^a^	21.42	−12.43	−33.86 (−49.67 to −18.04)	<0.001
Lipid measures at baseline, mean (SD), mmol/L				
LDL cholesterol	1.80 (0.71)	2.50 (1.11)	NA	NA
LS mean change^a^	−6.48	−30.63	−24.15 (−36.18 to −12.12)	<0.001
HDL cholesterol	1.10 (0.24)	1.10 (0.28)	NA	NA
LS mean change^a^	3.66	2.27	−1.39 (−9.56 to 6.77)	0.738
Triglycerides	1.60 (1.27)	2.10 (1.66)	NA	NA
LS mean change^a^	8.79	−8.48	−17.27 (−26.85 to −7.69)	<0.001
Total cholesterol	3.40 (0.88)	4.3 (1.35)	NA	NA
LS mean change^a^	−7.66	−22.74	−15.08 (−21.53 to −8.63)	<0.001

Abbreviations: HDL, high-density lipoprotein; LDL, high-density lipoprotein; LS, least-squares.

^a^ ANCOVA model.

### Subgroup analyses

**FIGURE 5:**
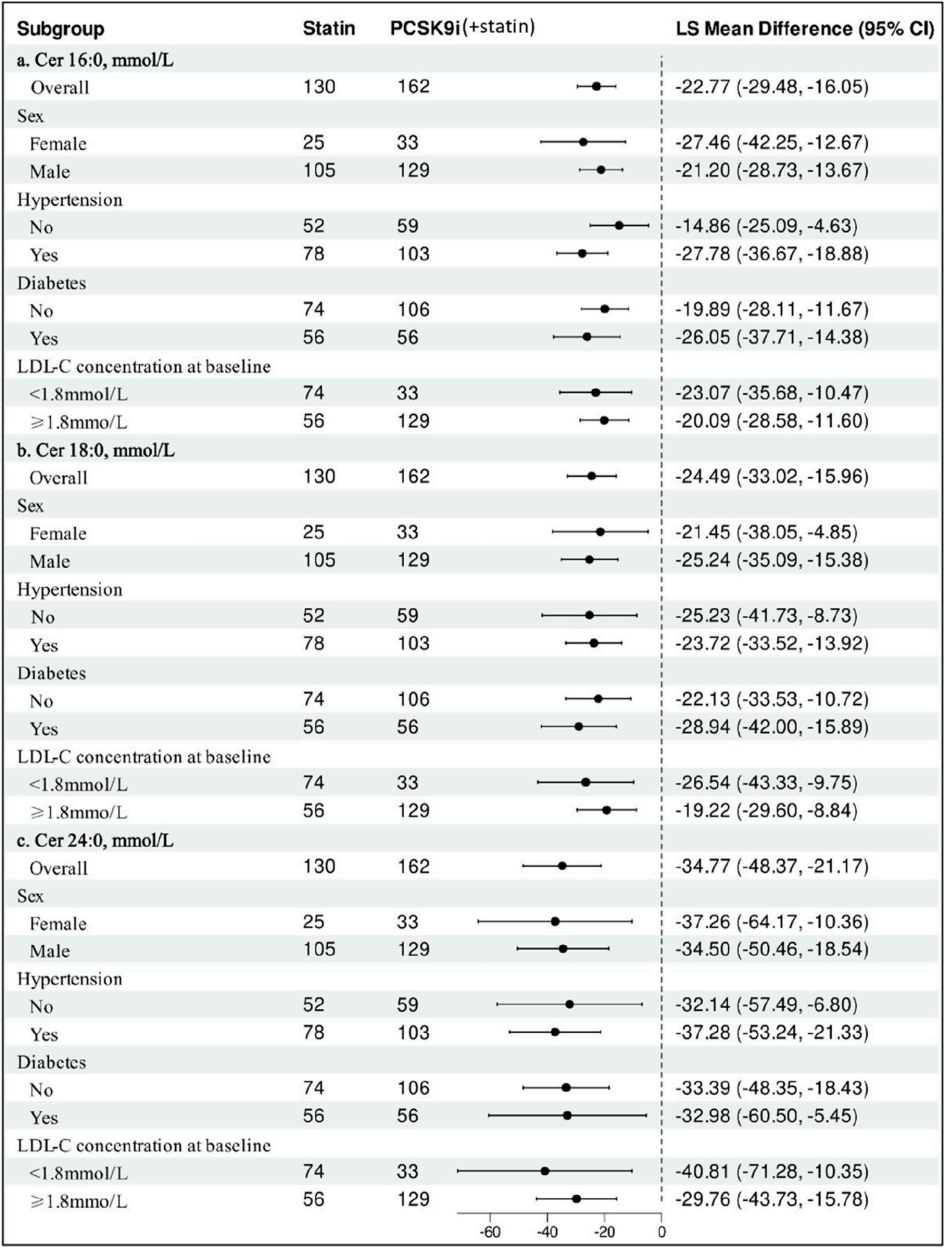
Subgroup Analysis of Statin-adjusted Percentage Change in Ceramide from Baseline to month with PCSK9i. Data are least-squares mean differences and 95% confidence intervals. The difference in the percentage change from baseline between PCSK9i and Statin was analyzed for each subgroup with the use of a ANCOVA model after adjusting age for repeated measures.

The impact of PCSK9i on change in ceramide among various subgroups is shown in Figure 5. The results remained consistent showing that PCSK9i was associated with a significant reduction of ceramide concentration.

## Discussion

This retrospective study firstly demonstrated that PCSK9i was further reduced ceramide levels beyond statins in the early stage of treatment. Our data shows that PCSK9 inhibitors reduced 3 distinct cardiovascular mortality associated plasma ceramide species that consistent with previous study (Ye et al., 2020; Hilvo et al., 2018). Cer 16:0, Cer 18:0 and Cer 24:1 decreased by 23%, 23%, and 19%, respectively. The change in Cer24:0, a protective or negative lipid species, respond differently to lipid-lowering therapy is a novelty finding. Cer 24:0 concentrations were significantly elevated in subjects treated only with statins, whereas treatment with PCSK9 inhibitors resulted in a significant reduction. Previously published study reported that treatment with PCSK9 inhibitors was associated with a 20%–40% reduction in Cer 24:0 (Ye et al., 2020; Hilvo et al., 2018). Additionally, the PCSK9 loss-of-function mutation (R46L) showed a 6.4% decrease in Cer 24:0, whereas simvastatin resulted in significant (25%) reductions, as reported in the study by Laaksonen (Jänis et al., 2013). The precise mechanisms are not clear, possibly due to the significant distribution of Cer 24:0 in the non-HDL lipoprotein fractions (Hilvo et al., 2018). Statin-adjusted reduction in Cer 16:0, Cer 18:0 and Cer 24:0 of up to 22.9%, 24.5%, and 34.8%, respectively, were also observed. The effects of PCSK9 inhibitors did not show a greater reduction in Cer 24:1 when administered alongside statins. Subgroup analyses showed that PCSK9i therapy significantly reduced ceramide concentration, regardless of the sex, LDL-C at baseline, or the presence of comorbidity (hypertension and diabetes). The change in LDL-C and other lipoprotein reported here was expected and is consistent with recent studies (Filippatos et al., 2018; Sabatine et al., 2017).

Lipid-lowering treatments reduce cardiovascular risk by less than one-third, which leaves many patients still susceptible to cardiovascular-disease events (Schwartz et al., 2018; Adhyaru and Jacobson, 2018). Therefore, achieving extreme reductions in LDL-C may not be necessary, and the benefit of lipid-lowering treatment may not be limited only to LDL-C (Tokgozoglu et al., 2025). Lp(a) levels were associated with atherosclerotic cardiovascular disease and risk of future cardiovascular events (Arnold et al., 2024). Recent meta-analyses from the 47 RCTs have demonstrated that an average of 27% Lp(a) reduction was achieved with PCSK9i therapy (Rivera et al., 2025). Furthermore, higher baseline Lp(a) levels are linked to a greater reduction in major adverse cardiovascular events and more significant Lp(a) reduction from PCSK9i (O'Donoghue et al., 2019; Bittner et al., 2020). Trials on cardiovascular outcomes with Lp(a) reduction using directly targeting Lp(a) therapies are still ongoing (NCT05563246, NCT05537571) (Nissen et al., 2024; Nicholls et al., 2025). However, there is no therapy specifically targeting the sphingolipid pathway.

Ceramides, biologically active molecular lipids, have causal roles in the process of LDL aggregation and significantly affect the pathogenesis of atherosclerosis (Edsfeldt et al., 2016; Park et al., 2004). The presence of elevated ceramide content in LDL particles may continue to signify a substantial cardiovascular risk despite having achieved the guideline-recommended LDL-C levels. Emerging data are suggesting that high ceramide may suffer substantial residual cardiovascular risk than LDL-C. Distinct plasma ceramide are key predictors of cardiovascular death in patients with stable CAD and acute coronary syndrome (ACS), but higher LDL-C levels did not correlate with an increase in high-risk patients (Laaksonen et al., 2016). Additionally, previous study found that patients with low LDL-C (<100 mg/dL) and high ceramides had a 16% annual incidence of cardiovascular events, compared to 3.7% for those with low LDL-C and low ceramides (Meeusen et al., 2018). Studies in rodent models reveal that alleviating inflammation and robust protective effects are achieved by reducing ceramide in the heart (Hadas et al., 2020). Targeting key enzymes in the ceramide metabolism shows promise as a therapeutic strategy in preclinical models (Choi et al., 2021). It is hence plausible to hypothesize that reducing ceramide levels may hold significant potential when combined with current interventions. Given the modest but significant reduction in ceramide levels, PCSK9 inhibitors may be used in the selection of CAD patients with higher ceramide levels for individualized treatments. Whether lowering ceramide and the extent of ceramide lowering required to reduces the risk of cardiovascular events is also not known.

### Potential mechanisms

The mechanisms relating changes in plasma ceramide with lipid-lowering therapy are not clear. Ceramides are produced in the liver and integrated into VLDL and LDL during its formation and release, a process partly facilitated by microsomal triglyceride transfer protein (Iqbal et al., 2015; Hammad et al., 2010). The reduction in ceramide may therefore reflect a decrease in the number of VLDL and LDL particles due to lipid-lowering therapy (Hilvo et al., 2018). Additionally, our findings indicate similar reductions in plasma Ceramide after adjusting for the LDL-C, which suggests that PCSK9i might lower plasma Ceramide through a different pathway beyond LDL-C lowering. Previous studies have demonstrated that the reduction in distinct sphingolipid species was similar for mice and humans with PCSK9 deficiency, suggesting a possible interaction between upregulation of the LDL receptor and circulating ceramide levels (Jänis et al., 2013).

## Limitation

Although we have carefully defined the time period for the repeated measurements to reduce confounding bias, the *post hoc* power analysis indicated excellent statistical power (>0.94) for detecting differences in all ceramide species reported in the current study. However, the study still has inherent flaws typical of retrospective research. A lack of information about statin use before admission, and information on use of antihypertensive drugs or diabetic medication, may interfere with the results. Additionally, sensitivity analysis and subgroup analysis were also consistent with the main analysis. Residual confounding by unmeasured or poorly measured factors, such as dietary changes and exercise intensity, may also impact changes in ceramide concentrations (Mathews et al., 2017; Reidy et al., 2020; Wang et al., 2017). The last concern is the imbalance of genders in the research sample. Larger studies, and longer exposure time are necessary to clarify whether changes in ceramide profiles affect the risk of progression of atherosclerosis and cardiovascular events.

## Conclusion

The present study demonstrate that PCSK9 inhibitors effectively decreased levels of distinct ceramides in patients with CAD while receiving statins. These findings strengthen the understanding of pathophysiologic mechanisms by which PCSK9 inhibitors affect sphingolipid pathway and have provided novelty insight for the design of drugs targeting the reduction of ceramides.

## Data Availability

The original contributions presented in the study are included in the article/Supplementary Material, further inquiries can be directed to the corresponding author.
