# Supplementary material for: Differential effects of PCSK9 inhibitors and statins on plasma ceramides in coronary artery disease
Source: Front Pharmacol. 2025 Dec 18;16:1726925. doi: 10.3389/fphar.2025.1726925 (PMC12756028; doi:10.3389/fphar.2025.1726925)
Supplement: Supplementary file 1 [file Table1.docx]

**Differential Effects of PCSK9 Inhibitors and Statins on Plasma Ceramides in Coronary Artery Disease**

Liang Zhang^1*^, Yaodong Ding^1*^, Yong Zeng^1^

1 Department of Cardiology, Beijing Anzhen Hospital, Capital Medical University, Beijing Institute of Heart, Lung and Blood Vessel Disease, Beijing, China

**Supplementary Material**

**STable 1. Demographic Characteristics before and after propensity score matching**

| **Characteristics** | **Before Matching** | | | **After Matching** | | |
| --- | --- | --- | --- | --- | --- | --- |
|  | **Statin** | **PCSK9i**  **(+statin)** | **SMD** | **Statin** | **PCSK9i**  **(+statin)** | **SMD** |
|  | **130** | **162** |  | **122** | **122** |  |
| **Male, n(%)** | 105 (80.8) | 129(79.6) | 0.029 | 101 (82.8) | 101(82.8) | <0.01 |
| **Hypertension, n(%)** | 78 (60.0) | 103 (63.6) | 0.073 | 74 (60.7) | 74 (60.7) | <0.01 |
| **Diabetes, n(%)** | 56 (43.1) | 56 (34.6) | 0.172 | 48 (39.3) | 48 (39.3) | <0.01 |
| **Stroke, n(%)** | 5(3.8) | 13(8.0) | 0.217 | 5(4.1) | 5(4.1) | <0.01 |

*SMD* Standardized Mean Difference.
