# Supplementary material for: Differential effects of PCSK9 inhibitors and statins on plasma ceramides in coronary artery disease
Source: Front Pharmacol. 2025 Dec 18;16:1726925. doi: 10.3389/fphar.2025.1726925 (PMC12756028; doi:10.3389/fphar.2025.1726925)
Supplement: Supplementary file 2 [file Image1.pdf]

Differential Effects of PCSK9 Inhibitors and Statins on Plasma Ceramides in Coronary Artery Disease

Liang Zhang[1*], Yaodong Ding[1*], Yong Zeng[1]

1 Department of Cardiology, Beijing Anzhen Hospital, Capital Medical University, Beijing Institute of Heart, Lung and Blood Vessel Disease, Beijing, China

Supplementary Material

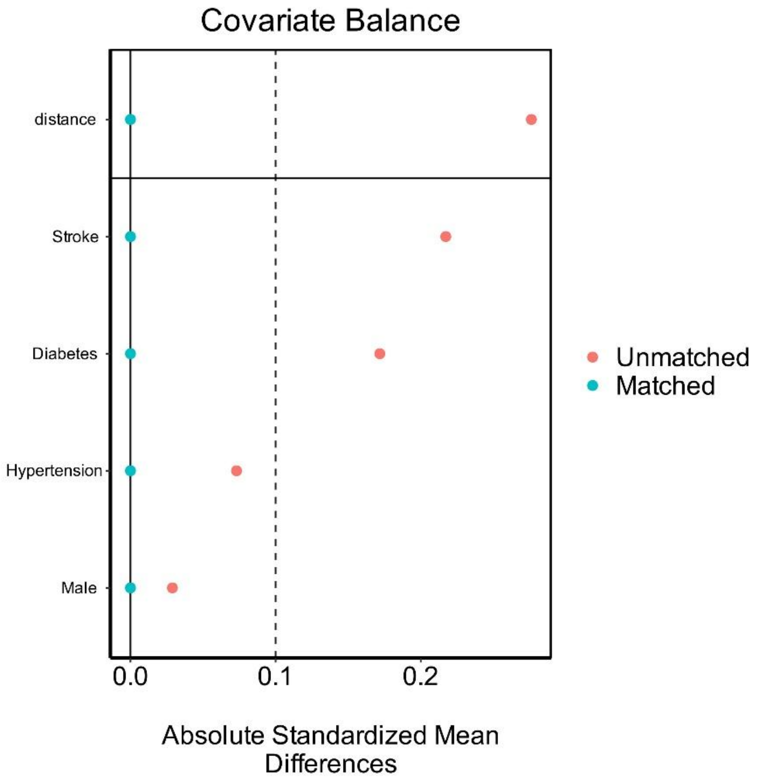

SFigure 1. Covariate balance before and after propensity score matching
